# Effect of COVID-19 pandemic restrictions on air pollution at a local scale in urban areas affected by high-intensity vehicle traffic in Poland

**DOI:** 10.1007/s11600-022-01005-0

**Published:** 2022-12-30

**Authors:** Beata Górka-Kostrubiec, Katarzyna Dudzisz

**Affiliations:** Institute of Geophysics, Polish Academy of Sciences, Księcia Janusza 64, 01-452 Warsaw, Poland

**Keywords:** Traffic-related pollution, Lockdown, Nitrogen oxides, Particulate matter, Warsaw, Cracow, Poland

## Abstract

The lockdown in 2020 implemented due to the SARS-CoV-2 pandemic has resulted in a significant improvement in air quality at a global scale. Nationwide lockdown also considerably improved air quality at a local scale, especially in cities which were almost completely shut down during the first coronavirus wave, with nearly no activity. We tested the hypothesis that a reduction in the intensity of vehicle traffic causes a drastic decrease in urban air pollution at a local scale. We focused on two urban agglomerations, Warsaw and Cracow, in Poland. Data of the concentrations of traffic-related sources, namely NOx, PM10, and PM2.5, obtained from two air pollution monitoring stations were analyzed for the years 2020 and 2021, during which lockdown and pandemic restrictions were in effect, and for 2019, as a reference. In the years 2020–2021, the average annual concentration of NOx was decreased by ~ 19%, PM2.5 by ~ 19%, and PM10 by ~ 18% in Warsaw, while in Cracow the average annual concentration of NOx was decreased by ~ 16%, PM2.5 by ~ 22%, and PM10 by ~ 2%, compared to 2019. The contribution from traffic-related sources to the overall level of air pollution was estimated. The results indicated that ~ 30 µg/m^3^ of PM10, ~ 15 µg/m^3^ of PM2.5, and ~ 120 µg/m^3^ of NOx in Cracow, and ~ 20 µg/m^3^ of PM2.5 in Warsaw originate from moving vehicles. The nationwide lockdown allowed us to conduct this study to understand how a reduction in local traffic emissions can decrease ambient air pollution levels.

**Supplementary Information:**

The online version contains supplementary material available at 10.1007/s11600-022-01005-0.

## Introduction

The epidemic caused by SARS-CoV-2 in the first half of 2020 has caused various changes in the activities of populations, which include maintaining a distance of at least 1.5 m between individuals, closure of public places (public and private offices, high and primary schools, etc.), avoidance of public meetings, and in consequence lockdown at a global scale.

Reports published in the last two years (Bar et al. 2021; Venter et al. 2020; Skirienė and Stasiškienė 2021; Girdhar et al. 2021; Filonchyk et al. 2020) emphasize that the economic slowdown caused by the coronavirus outbreak has caused a reduction in the global level of air pollution. A reduction in anthropogenic activities resulted in a significant improvement in air quality, mainly a decrease in the concentrations of nitrogen dioxide (NO_2_) and particulate matter (PM; particles smaller than 2.5 μm). Nitrogen oxides are a component in urban smog, which are known to be toxic to humans. NO_2_ is formed during the combustion of any fossil fuel in motor vehicles, thermal power plants, and manufacturing facilities (Li et al. 2018). NO_2_, a compound formed in the atmosphere by the oxidation of nitrogen oxide (NO), is the major cause of the formation of photochemical smog in cities with high-intensity vehicle traffic (Kamarehie et al. 2017). In Polish cities, more coal is used mainly in heating systems than in Western European countries; thus, the formation of NO_2_ from coal combustion should also be taken into account (Rogulski and Badydal 2021). Ultrafine PM composed of solid particles and liquid droplets (aerosols) is emitted into the atmosphere during high-temperature incomplete combustion processes and stack emission, as well as non-exhaust emission from cars. Both NOx and PM have the ability to penetrate deep into the lungs due to their small size and cause damage and thus have many harmful effects on humans.

Based on satellite measurements of air quality, Bauwens et al. (2020) estimated that NO_2_ pollution decreased by an average of 40% in Chinese cities and by 20–38% in Western Europe (Italy, Spain, France, Germany) and the USA during the 2020 lockdown, as compared to the previous year. Girdhar et al. (2021) indicated that in 44 cities in Northern China the levels of PM10 and PM2.5 decreased on average by 14% and 30%, respectively. These authors pointed out that a decrease in air pollution as a result of the lockdown has led to saving a few percent of human lives, i.e., by reducing the mortality rate attributed to air pollution from 7.6 to 3.4% (Isaifan 2020). In a study conducted in China, the growth rate of pollution over 60 years from 1950–1955 to 2010–2015 was recorded. The results revealed that due to the national lockdown announced on January 23, 2020, which lasted until March when manufacturing sectors gradually improved, a 70% reduction in NO_2_ was observed in areas with very intensive anthropogenic activities (Fan et al. 2020). As emphasized by Girdhar et al. (2021), this short-term slowdown of anthropogenic activities, mainly industries, provided an opportunity for China to become more breathable.

According to a report published by the Barcelona Institute for Global Health (ISGlobal, www.isglobal.org), the levels of air pollution to which approximately 84% of European citizens are exposed exceeded the limit proposed by the World Health Organization. The highest number of premature deaths related to NO_2_ pollution in Europe was reported in Madrid, Spain, whereas the second-highest number in Europe was recorded in Antwerp, Belgium. The third place was occupied by Turin, Italy, and the fourth place by Paris. In Poland, the highest number of NO_2_-related deaths has been reported in Warsaw. However, Polish cities are faring substantially worse in the ISGlobal ranking in terms of PM10 and PM2.5 pollution. The most polluted city is Brescia, Italy, where the highest number of fatalities related to fine PM (PM2.5) was recorded, while Warsaw occupied 20th place and 28th place on the list was Cracow.

Many publications indicate that the lockdown in 2020 implemented due to the SARS-CoV-2 pandemic caused a significant improvement in air quality at a global scale, for example, in Europe (Filonchyk et al. 2020; Gama et al. 2021; Higham et al. 2021; Wiśniewski et al. 2021; Ródenas et al. 2022; Baldasano 2020; Skirienė and Stasiškienė 2021; Jakovljevoć et al. 2021; Bar et al. 2021; Rogulski and Badyda 2021; Mikulski et al. 2021), USA (Bar et al. 2021), Canada (Mashayekhi et al. 2021), Mexico (Kutralam-Muniasamy et al. 2021), and China (Xu et al. 2020; Bauwens et al. 2020). This short-term improvement in air quality has a positive impact on human health in terms of reducing respiratory and other diseases, saving many thousands of lives, as annually approximately 7 million deaths occur due to global air pollution (WHO 2018). Furthermore, the lockdown in 2020 seemed to have improved the air quality even at a local scale, especially in cities that were almost completely shut down during the first coronavirus wave, with nearly zero activity (empty streets, traffic restrictions, etc.). In order to prevent SARS-CoV-2 infection, other European countries, including countries neighboring Poland, declared a state of emergency or place their countries under lockdown and stay-at-home orders. In the Czech and Slovak Republics and Lithuania, nationwide lockdowns were enacted in these countries on March 16, 2020. Schools and universities, nonessential stores, and all event venues were closed and restrictions on crossing state borders were introduced. In Germany, restrictions related to the SARS-CoV-2 pandemic were announced on March 22, 2020, and the administrations of the German federal states recommended staying at home. In Ukraine, the emergency was declared on March 20, 2020, while Belarus had not initiated a nationwide quarantine effort until 30 March. On 25 March, a mandatory 14-day self-quarantine requirement was instituted for persons entering Belarus from countries affected by the pandemic, and on 4 April, the government announced a two-week extension of spring vacation for schools.

In this study, we tested the hypothesis that a reduction in the intensity of vehicle traffic, mainly passenger cars, causes a substantial decrease in urban air pollution at a local scale. The nationwide lockdown period and pandemic restrictions that were in force for almost two years allowed us to conduct this study to understand how a reduction in local traffic emissions can decrease ambient air pollution levels. The local air quality is the most significant because it has the greatest as well as direct impact on the health of residents, especially in the most densely populated areas of cities.

For testing our hypothesis, we chose two large urban agglomerations in Poland—Warsaw and Cracow—in the central part of Europe. Data of step-to-step concentrations of three pollutants (NOx, PM10, and PM2.5), collected from two air pollution monitoring stations, were analyzed for 2020, when a lockdown was in effect, and for 2019, before the pandemic started. In addition, our research was extended to 2021, during which various pandemic-related restrictions were in force. The air pollution monitoring stations that provided the data are located in the central part of the cities of Warsaw and Cracow and monitor mainly traffic pollution. NOx concentrations were assumed to reflect exhaust emissions from moving vehicles, and PM concentrations to reflect non-exhaust emissions. While Warsaw and Cracow have some of the higher intensities of vehicle traffic in Poland, 2020 has seen the largest decrease (~ 9%) compared to previous years (2017–2019).

## Materials and methods

### Study area

Warsaw, the capital city of Poland, has about 1.8 million population (density of population about 3,470 people per 1 km^2^) and is located in the central part of the country in Mazovia. Cracow is located in Southern Poland, on the Vistula River, at the confluence of the Carpathian Mountains and Polish Uplands, and has about 767,000 inhabitants (density of population about 2370 people per 1 km^2^) (www.stat.gov.pl). In the central districts of Warsaw, air quality is mainly affected by traffic-related pollution with a relatively lower contribution of low-stack emission (from individual heating systems) in the winter season. Apartments in Warsaw have a central heating system, which mainly belongs to the large heat and power plant protected with electrostatic filters to limit the ingress of coarse PM into the atmosphere. In Cracow, domestic heating (furnaces or staves) is a significant source of PM during the winter season due to local and distant emissions emerging from individual heating systems from the use of coal and wood, and due to local environmental conditions such as air temperature inversions or katabatic flows causing even minor emissions to contribute to higher PM concentrations (Ścibor et al. 2020). Air pollution in the summer season (June–August) is mainly attributed to vehicle emissions.

### Data sources

**Fig. 1:**
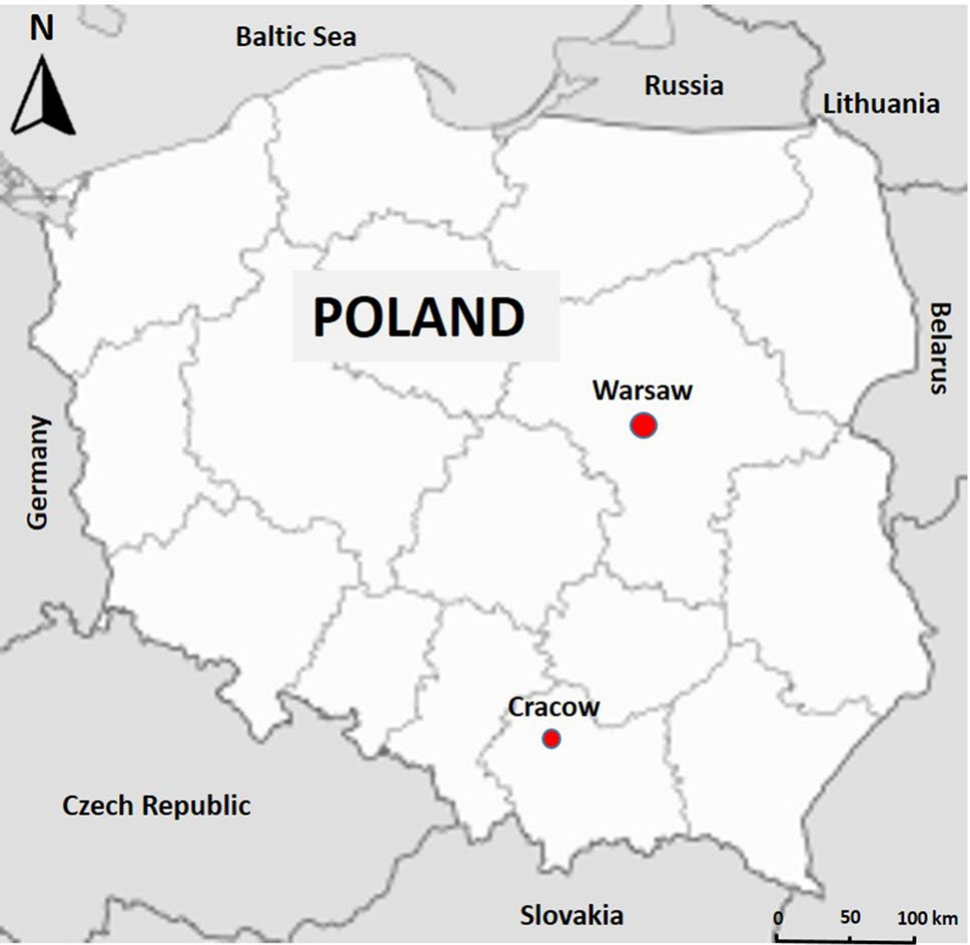
Map of Poland with sampling locations; Warsaw and Cracow

Data from two stations belonging to the national air quality monitoring network in Poland (Chief Inspectorate for Environmental Protection, http://powietrze.gios.gov.pl/pjp/archives) were analyzed (Fig. 1). In Table S1 (Supplementary material) are listed the stations in Warsaw and Krakow belonging to the national air quality monitoring network, with their attributes, i.e., country code, location, type of station, type of area, type of measurement, and type of monitored pollutants. The first studied station is located at Niepodległości Avenue in Warsaw (Traffic Station—Warsaw, TS-W), and monitors mainly traffic pollution. The Traffic Analysis in 2019 of the Warsaw Municipal Road Authority revealed that the average daily traffic in Niepodległości Avenue is about 70,000 vehicles (https://zdm.waw.pl/dzialania/badania-i-analizy/analiza-ruchu-na-drogach/). The second studied station is located at Krasiński Avenue in Cracow (Traffic Station—Cracow, TS-C), which also monitors traffic pollution. The average daily traffic density in Krasiński Avenue is approximately 73,000 vehicles (Report from traffic volume measurements at the entrances to Cracow commissioned by the Municipality of Cracow).

We analyzed the hourly concentration data of NOx (NOx = NO_2_ + NO), PM10, and PM2.5 for the months of 2019, 2020, and 2021. In Poland, the lockdown was first introduced on March 15, 2020, which was then tightened on March 25 and March 31, while the restrictions began to ease from April 24, 2020. Therefore, data on the average diurnal concentrations of all three pollutants for the period from February 1, 2020, to May 1, 2020, and data for the corresponding period of 2019 for comparison, were selected for the analysis. Additionally, we compared the monthly average concentrations of NOx, PM10, and PM2.5 in 2020 with that of the corresponding months in 2019 (before the SARS-CoV-2 pandemic), and the concentrations recorded in 2021 when the restrictions were partially removed and the urban community and economy partially returned to prepandemic conditions. The monthly and annual concentrations of pollutants were determined by averaging the daily values.

## Results

### Data analysis for Warsaw

**Fig. 2:**
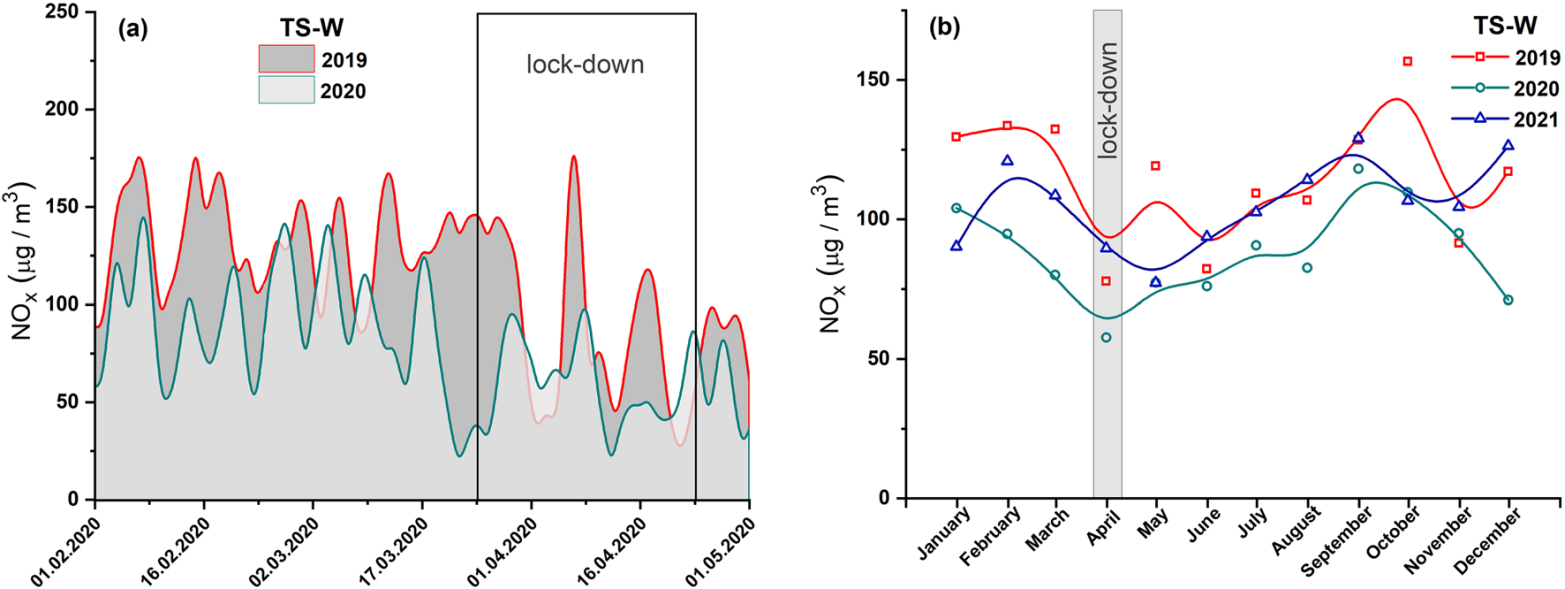
**a** Average diurnal concentrations of NOx prior to and after a lockdown in 2020 (light area) and comparison for the same period in 2019 (dark area). **b** Average monthly concentrations of NO_x_ in 2021, 2020, and 2019. Data from air pollution traffic monitoring station (TS-W), Warsaw, Poland. B-spline curve (basis spline function) was applied for the smoothness of experimental data

Figure 2a presents the average diurnal concentrations of NOx prior to and after the lockdown in 2020, in comparison with the data obtained for the same period in 2019. A significant decrease was observed in the diurnal level of NOx starting from March 25, 2020 (the beginning of the lockdown), which continued further (Fig. 2a). Some peaks in the level were still noted (up to May 1, 2020), but their intensity was significantly lower than that before the lockdown. A downward trend was also seen during the lockdown in the monthly average concentrations of NOx (ca. 57.7 µg/m^3^) for all months surveyed between 2019 and 2021 (Fig. 2b), and the values dropped by about 26% and 36% in comparison with 2019 and 2021, respectively. The monthly average NOx concentration of each year showed a similar trend, which can be probably related to seasonal changes. In the winter season (from December to March), the NOx values were high and then declined to reach a minimum in April (2019 and 2020) and May (2021) and again increased in the summer and fall seasons (Fig. 2b). A significant decrease in the level of NOx air pollution was also seen for the whole of 2020 in comparison with 2019 and 2021 (Fig. S1a in Supplemantary material).

**Fig. 3:**
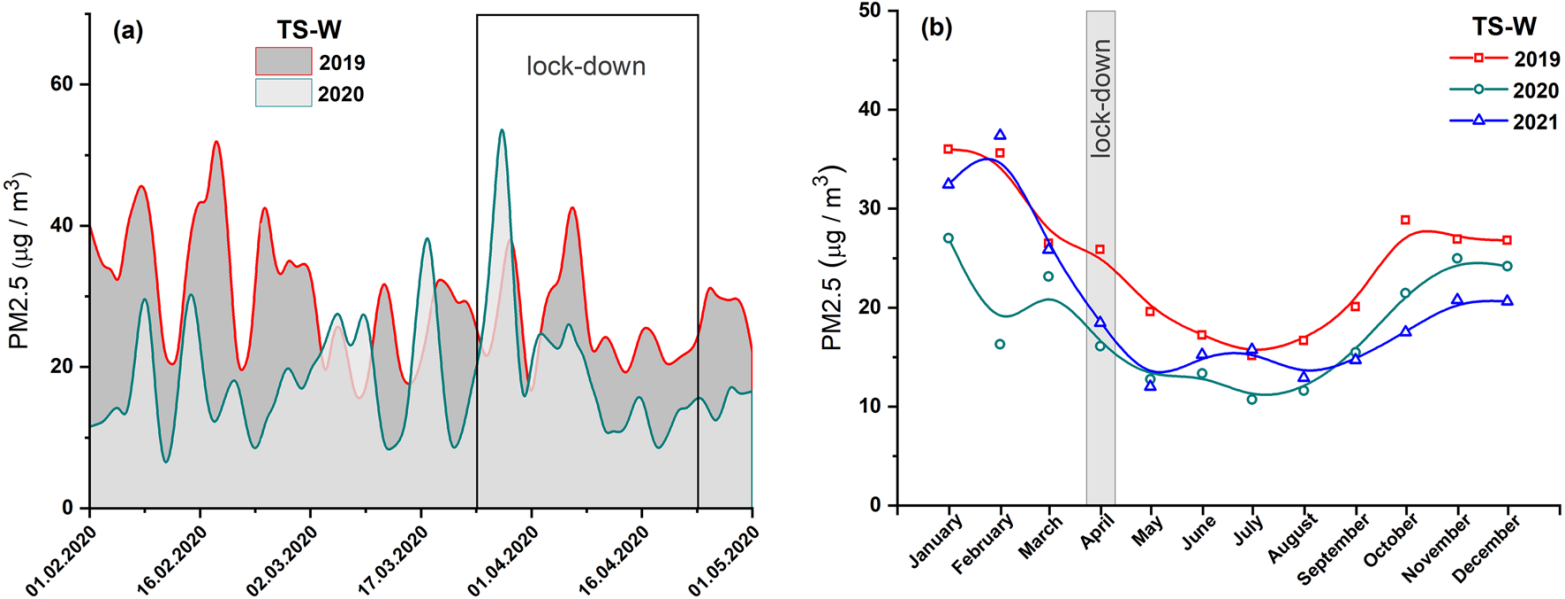
**a** Average diurnal concentrations of PM2.5 prior to and after a lockdown in 2020 (light area) and comparison for the same period in 2019 (dark area). **b** Average monthly concentrations of PM2.5 in 2021, 2020, and 2019. Data from air pollution traffic monitoring station (TS-W), Warsaw, Poland. B-spline curve (basis spline function) was applied for the smoothness of experimental data

**Fig. 4:**
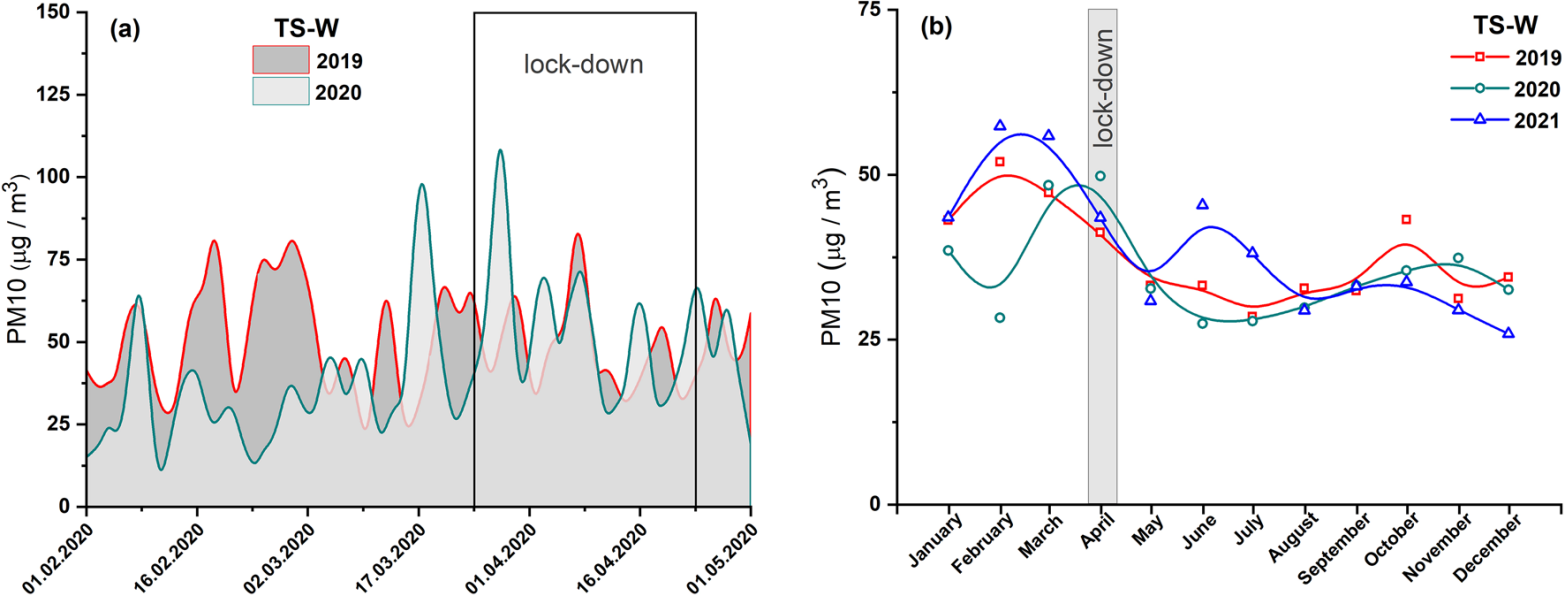
**a** Average diurnal concentrations of PM10 prior to and after a lockdown in 2020 (light area) and comparison for the same period in 2019 (dark area). **b** Average monthly concentrations of PM10 in 2021, 2020, and 2019. Data from air pollution traffic monitoring station (TS-W), Warsaw, Poland. B-spline curve (basis spline function) was applied for the smoothness of experimental data

The average diurnal and monthly concentrations of PM2.5 and PM10 before and after the lockdown in 2020, and for comparison, the data for the same period in 2019 are presented in Figs. 3a and 4a, respectively. No significant decrease in the concentrations of both types of PMs was observed during the lockdown. The peaks in PM10 (Fig. 4a) and PM2.5 (Fig. 3a) concentrations during the lockdown in 2020 were of higher intensities than those observed before March 25, 2020. This is well reflected by the monthly average values of PM10 shown in Fig. 4b. In April 2020, the concentrations of PM10 were higher than that recorded for the same month in 2019 (by ~ 21%) and 2021 (by ~ 14%). On the other hand, the monthly average PM2.5 concentrations (Fig. 3b) in April 2020 were lower by 38% and 13% compared to the same month in 2019 and 2021, respectively. The level of PM10 was not influenced by lockdown-induced changes in residents’ activity (staying at home, home office) and the resulting restrictions on travel by private cars and public transport.

The weather data from the publicity available repository of the Polish Institute of Meteorology and Water Management were analyzed. The weather data for two months March and April in 2019, 2020, and 2021 are listed in Table S2 (Supplementary material). The monthly average temperatures were 5.0 °C and 9.5 °C in March and April 2020. In March and April same year, the monthly humidity and wind speed were 59% and 53%, and 4.1 m/s and 3.2 m/s, respectively. March and April 2020 are characterized by changing wind direction, relatively low precipitation, with monthly average values of 13.0 mm in March and 7.5 mm in April.

### Data analysis for Cracow

**Fig. 5:**
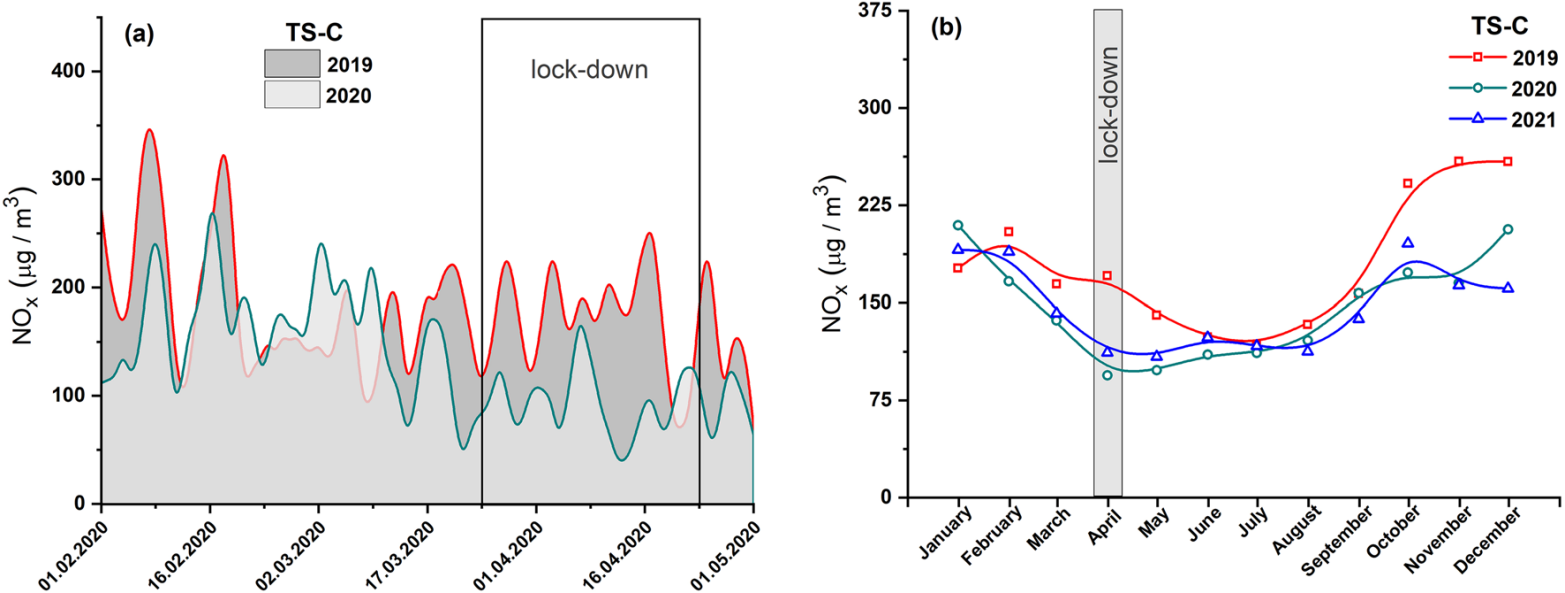
**a** Average diurnal concentrations of NOx prior to and after a lockdown in 2020 (light area) and comparison for the same period in 2019 (dark area). **b** Average monthly concentrations of NO_x_ in 2021, 2020, and 2019. Data from air pollution traffic monitoring station (TS-C), Cracow, Poland. B-spline curve (basis spline function) was applied for the smoothness of experimental data

The analysis of temporal variations in the concentration of NOx in Cracow showed a visible decrease after the lockdown implemented on March 25 (Fig. 5a). This downward trend was particularly well reflected by the monthly average concentrations of NOx shown in Fig. 5b. The lowest NOx concentrations (~ 94.3 µg/m^3^) were recorded in April 2020 among all the months from 2019 to 2021. Compared to 2019, a 45% decrease in NOx concentrations was observed in April 2020, while in the corresponding month in 2021 the decrease was approximately 16%. In each year, the concentrations of NOx varied based on seasonal changes. The concentrations were high during winter (from December to February) and then decreased reaching a minimum in April 2020 and 2021, and in July 2019, followed by an increase during summer and fall. Interestingly, however, a significant decrease in NOx air pollution levels was observed throughout 2020 and 2021 compared to 2019 (Fig. S1b in Supplemantary material).

**Fig. 6:**
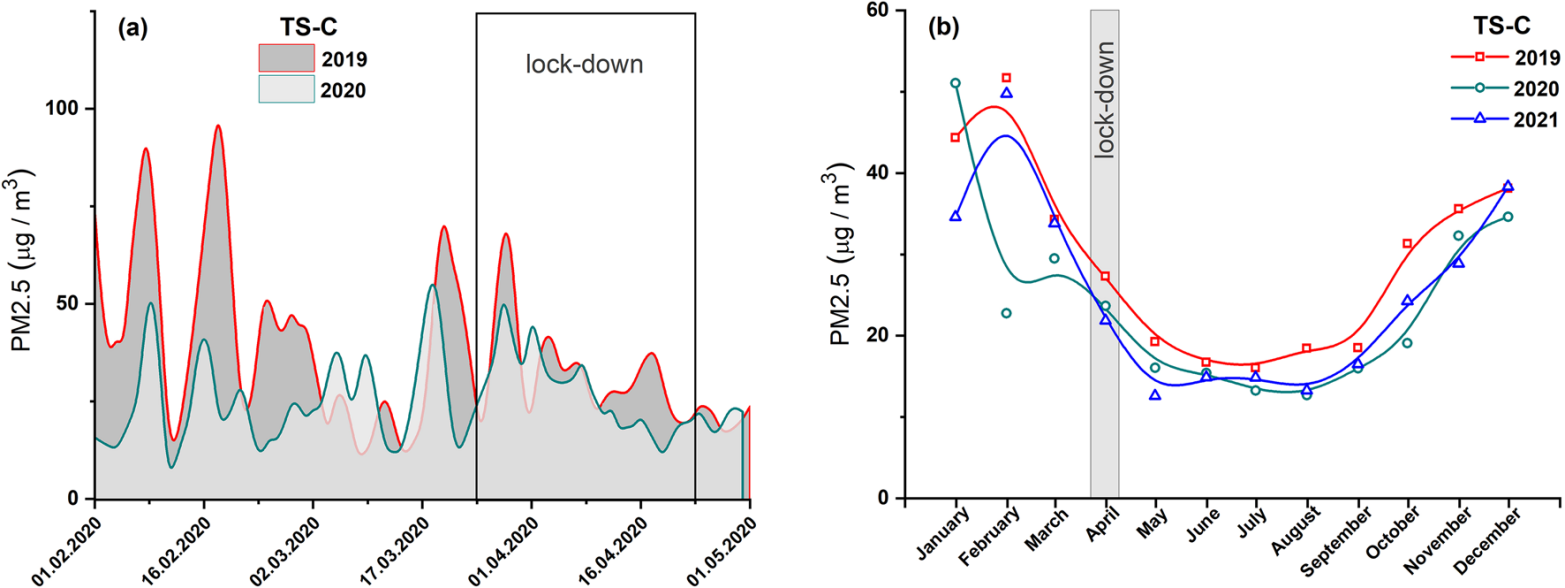
**a** Average diurnal concentrations of PM2.5 prior to and after a lockdown in 2020 (light area) and comparison for the same period in 2019 (dark area). **b** Average monthly concentrations of PM2.5 in 2021, 2020, and 2019. Data from air pollution traffic monitoring station (TS-C), Cracow, Poland. B-spline curve (basis spline function) was applied for the smoothness of experimental data

**Fig. 7:**
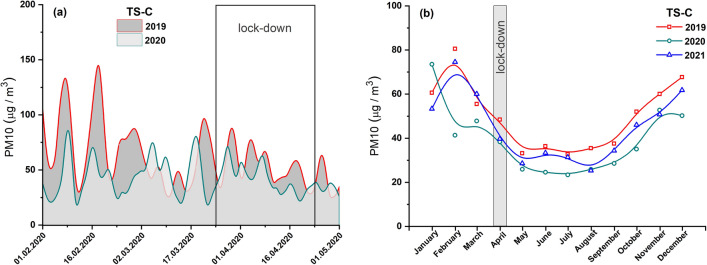
**a** Average diurnal concentrations of PM10 prior to and after a lockdown in 2020 (light area) and comparison for the same period in 2019 (dark area). **b** Average monthly concentrations of PM10 in 2021, 2020, and 2019. Data from air pollution traffic monitoring station (TS-C), Cracow, Poland. B-spline curve (basis spline function) was applied for the smoothness of experimental data

The concentrations of PM2.5 and PM10 did not significantly decrease during the full lockdown period, i.e., from March 20, 2020, to April 24, 2020 (Figs. 6a and 7a). The monthly average PM10 concentrations in April 2020 reduced by 21% compared to 2019 and by 3% compared to 2021 (Fig. 7b), while that of PM2.5 declined by 13% compared to 2019 and increased by 8% compared to 2021 (Fig. 6b). On the other hand, the annual average PM concentrations in 2019, 2020, and 2021 did not show significant changes (Fig. S2 in Supplemantary material).

The weather data for two months, March and April in 2019, 2020, and 2021 are listed in Table S2 (Supplemantary material). The monthly average temperatures were 4.9 °C and 9.5 °C in March and April 2020. In March and April of the same year, the monthly humidity and wind speed were 65% and 52%, and 2.1 m/s and 1.3 m/s, respectively. The dominant wind direction in March 2020 is from west and east, whereas in April 2020, wind was blowing mainly from the west. March and April 2020 are characterized by relatively low precipitation, and the monthly average values were 15.2 mm in March and 4.4 mm in April.

### Air pollution related to traffic intensity

The data of average vehicle traffic intensity recorded at 33 sites across Poland by the General Directorate for Roads and Highways (GDDiA) show that the average daily traffic intensity of all vehicles in total in April 2020 was 42% lower than that of the same month in 2019, while the values observed at individual sites ranged from 30 to 60%. These differences mainly arise from a decrease in the proportion of passenger vehicles in the total number of moving vehicles due to restricted travel during the lockdown period. Moreover, in April 2020, passenger and light vehicle traffic lacked the characteristic Friday peak. On the other hand, a significant decrease in traffic volumes was observed on the weekends, compared to the standard weekly traffic schedules (General Directorate for Roads and Highways, Report—General Measurement of Traffic 2020/2021, www.archiwum.gddkia.gov.pl). As absolute data for the daily traffic density of vehicles on the streets of Warsaw and Cracow are not available, we used the data of average traffic congestion level (TCL) (Table 1) published at https://www.tomtom.com. A congestion level (in %) indicates how much longer a trip will take than it would under baseline uncongested conditions. The baseline for each city is calculated by analyzing free-flowing travel times of all vehicles on the entire road network that are recorded 24/7, 365 days a year. Calculations are performed for all hours of each day, to determine congestion levels at any time in any city, including morning and evening peak hours.

**Table 1:** Average traffic congestion levels (TCL) in 2021, 2020, and 2019, and their changes compared to the previous year for Warsaw and Cracow cities (https://www.tomtom.com after traffic index ranking)

Year	Warsaw	Cracow
2021	35% (increased by about 4%)	41% (increased by about 5%)
2020	31% (decrease by about 9%); April 2020 (during lockdown) decrease by about 13%	36% (decrease by about 9%); April 2020 (during lockdown) decrease by about 14%
2019	40% (increased by about 1%)	45% (increased by about 5%)

Changes in the average TCL compared to the previous year are shown in brackets

**Fig. 8:**
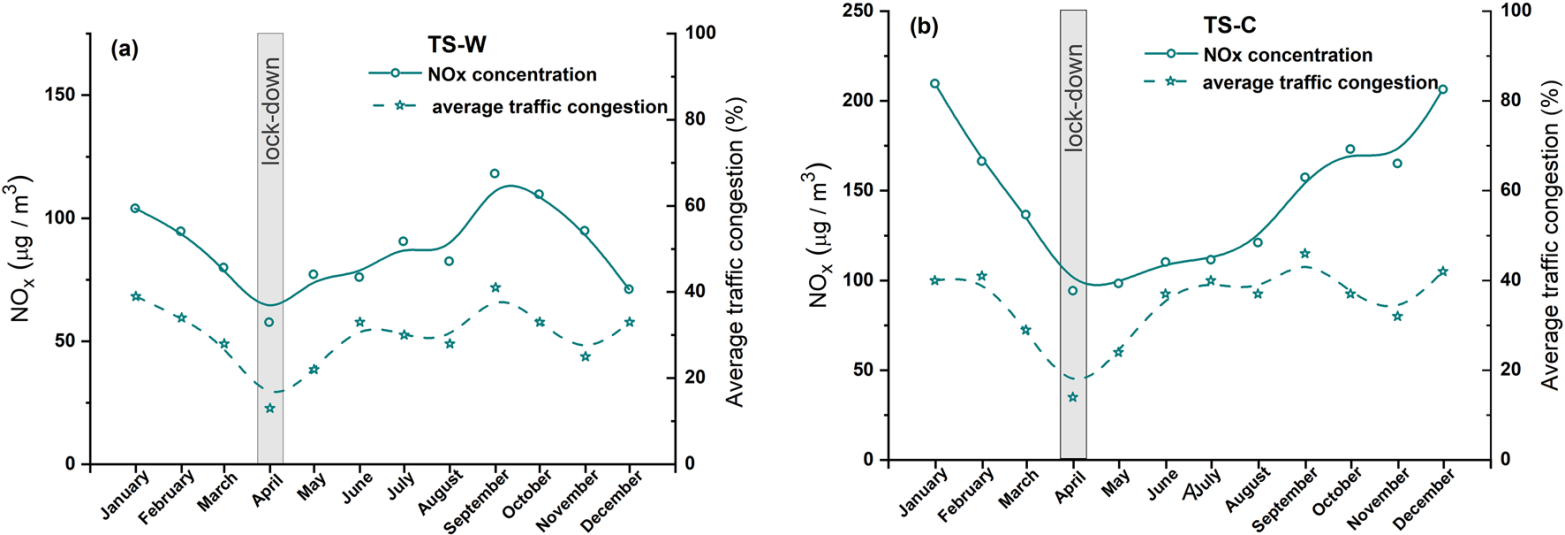
Average monthly NOx concentration (open circles) and average monthly traffic congestion level (TCL) (open stars) in 2020 in the study area TS-W, Warsaw (**a**) and TS-C, Cracow (**b**). B-spline curve (basis spline function) was applied for the smoothness of experimental data

The correlations between pollutant concentrations and intensity of vehicle traffic, expressed as the average TCL of each month in 2020 (Table 2), at the studied locations in Warsaw and Cracow were analyzed in detail. The results showed significant correlations only for the concentrations of NOx at both studied locations. The calculated Pearson correlations between the NOx concentrations and TCL were 0.75 and 0.6 for Warsaw and Cracow, respectively. Both PM fractions at the Cracow site were poorly correlated with TCL (correlation coefficient lower than 0.04). In the case of Warsaw, a weak correlation, with a coefficient of 0.22, was observed for PM2.5, while a negative correlation was noted for PM10. Thus, it appears that exhaust traffic-related emissions may be best reflected by NOx concentrations. Consequently, a relationship between the average monthly NOx concentration and the average monthly TCL was found for Warsaw (Fig. 8a) and Cracow (Fig. 8b). A significant reduction in the concentration of NOx was observed during the full lockdown (in April). This may indicate that a reduction in the number of motor vehicles on the roads of the city contributes to an improvement in air quality on a local scale, as well as on a global scale.

**Table 2:** Average TCL of each month in 2020 in Niepodległości Avenue (TS-W) in Warsaw and Krasiński Avenue (TS-C) in Cracow

Month in 2020	01 (%)	02 (%)	03 (%)	04 (%)	05 (%)	06 (%)	07 (%)	08 (%)	09 (%)	10 (%)	11 (%)	12 (%)	Average (%)
TS-W	39	34	28	13	22	33	30	28	41	33	25	33	30
TS-C	40	41	29	14	24	37	40	37	46	37	32	42	35

## Results interpretation and discussion

### Impact of lockdown on pollution levels

Of the three pollutants NOx, PM10, and PM2.5 measured at traffic pollution monitoring stations, two of them (NOx and PM2.5) were identified as good indicators of local-scale air pollution levels influenced by traffic-related urban activities. During the full lockdown, the concentrations of both pollutants significantly decreased due to restrictions on travel by private and public transport. Most studies (e.g., Tian et al. 2021; Gama et al. 2021; Chen et al. 2021) indicated a relationship between transport flow and air pollution caused by NOx. Our study showed that motor vehicle exhaust emission has the greatest impact on NOx levels at a local scale. Both the investigated cities—Warsaw and Cracow—experienced a decrease in the local NOx levels during the lockdown period. However, the observed decrease seemed to be more significant in Cracow (by ~ 45%) than in Warsaw (by ~ 26%). The smaller decrease in NOx concentration in Warsaw may be due to the disproportionately strong decrease in NOx concentration in April 2019 (Fig. 2b). In both cities, NOx air pollution increased in the post-lockdown period as people began to travel and perform their daily activities. In Cracow, a continuous increase in the NOx level was observed till December 2020 (Fig. 5b), and the concentrations in July and August 2020 were as high as in the corresponding months of 2019. In Warsaw, an increase in NOx concentration was observed until October, followed by a decrease in November and December 2020 (Fig. 2b). This decline may be attributed to the second coronavirus wave in Poland and the renewal of government restrictions in those months.

PM2.5 can be considered as an indicator of local-scale air pollution caused by non-combustion emissions. This type of fine particle is generated by vehicle circulation-related processes, such as abrasion of brake pads and disks, pavement, and vehicle tires. PM has the greatest impact on health leading to the development of cardiovascular diseases and chronic obstructive pulmonary disease, among others. During the lockdown (April 2020), PM2.5 concentrations decreased by 38% and 13% in Warsaw and Cracow, respectively, compared to 2019. However, these concentrations during the lockdown period were not the lowest in the entire 2020 and steadily decreased in subsequent months reaching minimum values in summer (July–August). This may indicate that sources that dominate the winter season slightly contribute to PM2.5 concentrations.

The European Space Agency satellite imagery revealed a marked change in global NOx levels (https://www.esa.int/Applications/Observing_the_Earth/Copernicus/Sentinel-5P/Coronavirus_lockdown_leading_to_drop_in_pollution_across_Europe) during the lockdown. Using simulation models, Menut et al. (2020) estimated a 30–50% decrease in the concentrations of NO_2_ and PM in all Western European countries during the 2020 lockdown. The average decrease in NO_2_ in European cities ranged between 27 and 25% (Bar et al. 2021; Bauwens et al. 2020). A substantial reduction in NOx emissions was apparent in different Italian cities, especially Lombardy and Veneto as well as Turin and Bologna (Bauwens et al. 2020). In Milan and Venice, the average tropospheric concentration of NO_2_ during the lockdown in 2020 decreased by about 38% than during the same period in 2019. A marked decline in the NO_2_ column, by about 30%, was also observed during the strict lockdown in Spain and France in comparison with the same period in 2019. A relatively moderate decrease of about 20% was observed in Germany and Belgium, which can be due to less strict lockdown restrictions in these countries (Bauwens et al. 2020). At the traffic site in Zagreb, Croatia, the concentrations of NO_2_ decreased by about 35% during the COVID-19 lockdown in comparison with that in the same period in the previous year (Jakovljevoć et al. 2021). In five Polish cities, the global concentrations of PM2.5, PM10, and NO2 decreased during the lockdown period. The data acquired from air quality monitoring stations in various areas of the city, including urban, suburban, rural, and industrial zones, indicated a reduction by 11.1–26.4%, 8.6–33.9%, 18–23%, and 10–19%, respectively, compared to the corresponding periods in 2018 and 2019 (Filonchyk et al. 2020). A 20% reduction in PM2.5 concentration was recorded in April in Warsaw and an 11.1% reduction in Cracow as compared to the same period of 2019. For PM10, a reduction by 11.8% and 8.6%, was observed in April in Warsaw and Cracow, respectively. An even greater reduction was observed in the NO_2_ levels in April, and the recorded decrease was 8.6% in Warsaw and 20.7% in Cracow as compared to 2019. Our study showed that in both cities reductions in the levels of NOx and PM2.5 during the full lockdown in April 2020 were significantly higher in comparison with those reported by Filonchyk et al. (2020). This is because we analyzed the data on the local level of pollution obtained from stations that monitor mainly traffic-related pollution. At other air pollution monitoring stations classified as urban (no traffic), the decrease in NOx concentration in April 2020 compared to the corresponding month of 2019 was 10–12% and 1–7% for Warsaw and Cracow, respectively.

Air quality in the area affected by traffic sources may also be influenced by weather conditions, such as movements of air masses (wind speed and direction), humidity, and temperature (Mikulski et al. 2021). In Table S2 (Supplemantary material), the average values of main meteorological parameters for March and April 2019, 2020, and 2021 are given for both studied cities (Warsaw and Cracow). We state that temperature can affect the level of air pollution mainly by increasing the demand for thermal energy in individual and central heating systems. The average temperature values in March and April 2020 did not differ significantly from the same months in 2019 (see Table S2 Supplemantary material); however, concentrations of pollutants do not reflect this trend during the lockdown. This indicates that the reduction in pollution levels may be mainly due to lockdown and limitation of the mobility of inhabitants. Comparing pollutant concentration data with the average amount of rainfall, no relationships were found that could explain the leaching of pollutants from the atmosphere. For example, in March and April 2020 lower average precipitation values were recorded in Cracow compared to the same months of 2019, while concentrations of NOx, PM10 and PM2.5 were the highest. We state that the wind speed may be the main factor that contributes to the removal of pollutants (ventilation of the city). In Cracow, the average wind speed does not exceed 2.1 m/s, while in Warsaw it is more than 1 m/s higher than in Cracow. This may be one of the reasons for the relatively higher levels of air pollution recorded in Cracow compared to Warsaw during the lockdown.

The national lockdown allowed us to conduct this study to determine air quality in cities on a local scale. This enabled us to understand how the reduction of traffic intensity and stack emissions can affect the levels of air pollution in our closest surroundings. Our research clearly showed that the local pollution in the areas of traffic-affected cities can be significantly higher than the average level observed for the entire city. This information may be useful to people living (working or studying) in these areas because they are exposed to significantly or even several-fold higher levels of NOx and fine PM. This, in turn, may more frequently lead to health effects, such as respiratory infections or inflammation, and in the worst case, even cancer due to prolonged exposure to high levels of NOx and fine PM fractions. A detailed study allows identifying the factors that may reduce our exposure to air pollution on a local scale. Some of the sources of air pollution such as traffic and low emissions can be mitigated and, to some extent, controlled. However, some sources are less controllable because they affect the levels of pollution on a larger scale, such as a city. This hinders achieving a significant reduction in exposure because the source of contamination cannot be avoided. For example, investigations conducted in Tokyo and Singapore have simultaneously recorded PM2.5 concentrations with the use of personal sensors (a portable air quality backpack) carried by people moving through both cities during the lockdown period and using a network of stationary outdoor air quality sensors (https://www.dyson.com.sg/newsroom/dyson-investigates). In Tokyo, the levels of PM2.5 recorded by personal sensors were consistent with the data collected by a network of outdoor stations. Both types of loggers recorded extreme values of pollution, which were linked to ongoing fires and increased pollen activity, as well as dust storms. By contrast, the data obtained from the outdoor sensor network and PM2.5 levels recorded by personal sensors in Singapore were not consistent. This suggests that the participants were influenced by more local pollution events.

### Correlations among pollutants

Studies analyzing whether and how strongly the pollutants correlate with particular months of the year and with each other are interesting. Therefore, we examined the correlations of the average monthly concentration of pollutants in the particular months of 2019, 2020, and 2021 and the correlations among pollutants in the years 2019–2021 (Tables S3 and S4 Supplemantary material). The obtained results were promising, although significant differences were observed in the activity of residents between 2019 and 2021. For Cracow, the correlation coefficients for the particular months of 2019, 2020, and 2021 were in the range of 0.52–0.89 for PM2.5, 0.64–0.90 for PM10, and 0.67–0.82 for NOx (Table S4 Supplemantary material). These values seem to reflect seasonal changes. The good and very good correlations of monthly average pollutant concentrations for 2019–2021 indicate that they mimic trends that depend on pollutants throughout the year, i.e., high concentrations in the cold half and low concentrations in the warm half of the year. These changes are characteristic of Polish cities, as in the cold months, the interiors of buildings must be heated by central or individual heating systems. Increased demand for thermal energy implies an increased emission of pollutants into the atmosphere. The report of the European Environmental Agency (EEA) (https://www.eea.europa.eu/) highlights that in Poland high levels of smog concentrations originate from home heating with the use of coal, which is often of very poor quality. This accounts for 80% of PM2.5 emissions entering our lungs. Neighboring countries prefer natural gas, which is much less hazardous to health, for home heating.

In Cracow, significant correlations have been observed between PM2.5 and PM10 (correlation coefficient 0.97–0.99) in every studied year. This suggests that both types of pollutants may originate from the same source and contribute in the same way to air pollution in Cracow every month. This part of PM appears to be mainly related to stack emission. As indicated by Ścibor et al. (2020), abundant air pollution in Cracow is an effect of the combination of local and distant combustion emissions, and local environmental conditions. Although about 90% of flats in Cracow are heated by the central heating system, exhaust emissions from domestic heating significantly contribute to PM, mainly during the winter season. It is estimated that Cracow is surrounded by over 21,000 households with an individual heating system burning mainly coal and wood and that about 15% of pollutants floating over Cracow during the heating season come from neighboring municipalities (Report #Oddychaj Polsko, www.airly.org). The spread of pollution in Cracow is influenced by local environmental conditions, such as air inversion temperature or katabatic flows (Ścibor et al. 2020). In such climatic conditions, even relatively small emissions can increase the concentration of pollutants close to the ground surface due to their poor dispersion into higher atmospheric levels and/or inability to blow them outside the city.

For Warsaw, the correlation coefficients calculated for the years 2019–2021 were as follows: 0.54–0.88 for PM2.5, 0.21–0.72 for PM10, and 0.36–0.64 for NOx (Table S3 Supplemantary material). Thus, the average monthly concentrations of PM10 and NOx seem to be less correlated in Warsaw than in Cracow. In particular, the weakest correlations were found between the concentrations of NOx in 2019 and 2021 (*r* = 0.36), and between the concentrations of PM10 in 2020 and 2021 (*r* = 0.27). The weak correlations observed between the NOx concentrations in 2019 and 2021 may be related to the low concentrations in January 2021, which were a continuation of the strong decline that already began in November 2020. This may be mostly due to government-implemented restrictions during the second wave of the coronavirus pandemic in Poland, which started in the fall of 2020 and lasted until January 2021. The restrictions that were in force were not as severe as during the first wave of COVID-19; however, schools adopted remote education as did some government offices and private companies. This entailed a reduction in the number of vehicles on the streets in Warsaw. In Cracow, the dominant stack emissions during the cold season may have masked the reduction of NOx emitted due to transport resulting from restrictions and limitations during the second wave of coronavirus in Poland. This is confirmed by the good correlations of the NOx concentrations with the concentrations of PM10 and PM2.5 in Cracow (0.67–0.75). For Warsaw, these correlations were weak (0.48–0.19), and even a negative correlation was found between the concentrations of NOx and PM10 in 2020.

### The distribution of pollutants in both cities

Our analysis indicated that the changes in residential activity in 2020 due to the pandemic had a real impact on the local level of air pollution associated with car transport. The annual average concentrations of PM2.5, PM10, and NOx in 2020 were nearly 26%, 6%, and 24% lower compared to that in 2019 as recorded by TS-W (Figs. 2, 3, 4, and and S1a), and 18%, 22%, and 18% lower as recorded by for TS-C (Figs. 5, 6, 7, and S1b). Based on the commonly accepted levels of pollutants in the air, especially PM with a particle size below 2.5 μm (annual average concentration 20 µg/m^3^), we can conclude that in the summer season (June–August), the permissible annual level of PM2.5 is 14.3 µg/m^3^ for Warsaw and 15.7 µg/m^3^ for Cracow. On the other hand, in winter, the concentrations of harmful PM2.5 particles were on average exceeded by 35% in Warsaw and 87% in Cracow.

As shown in Figs. S1a and S1b, the annual concentrations of all pollutants in 2020 were higher in Cracow’s traffic station than in Warsaw’s traffic station. This finding agrees well with the annual TCL (Table 2). In TS-C in Cracow, the annual TCL in 2020 was on average 5% (Table 2) higher than that in TS-W in Warsaw. Consequently, based on the data recorded for the lockdown period (April 2020), it can be deduced that a decrease in TCL of about 9% results in a decrease in local air pollution levels by about 45%, 21%, and 13% for NOx, PM10, and PM2.5, respectively, in Cracow’ traffic station. For Warsaw, the same decrease in TCL during lockdown was followed by a decrease in NOx of about 26% and in PM2.5 of about 38%, with an unexplained increase in PM10 of about 21%. The local increase in PM10 is not matched by global changes across the city (indicated by data cited earlier for Warsaw), which suggests that local sources or events emitting this type of PM into the atmosphere have a significant influence. The PM10 concentrations shown in Fig. 7 highlight that the PM10 concentrations in June and July 2021 were higher compared to the values observed in those months of 2019 and 2020. Skirienė and Stasiškienė (2021) and Han and Naeher (2006) pointed out that the assessment of the effect of the lockdown on PM10 changes is more complex than the identical analysis performed for NOx. PMs present in the air can originate from many sources. The concentration of PM10 may be affected by meteorological conditions as well as emission of primary PMs from a variety of sources, including traffic, industry, commerce, and domestic heating. It must be noted that when people had to stay at home during the restricted lockdown, they mostly used individual stoves/fireplaces to maintain warm conditions in their homes. As a result, primary PM emissions resulting from domestic combustion of coal or wood as well as central heating plants started to dominate, while the concentrations of NO_2_ and primary PM emissions from road traffic reduced (Chen et al. 2021; Coccia 2021). Consequently, a decrease in PM10 emissions related to road traffic could be compensated by an increase in PM10 arising from the combustion of coal and wood for domestic heating (Vultaggio et al. 2020; Collivignarelli et al. 2020). In the cities of Northern Italy, the lockdown did not cause any reduction in the concentrations of PM2.5 and PM10. On the contrary, the concentrations were higher by 24.1% and 20.9%, compared to the same period in 2019 (Skirienė and Stasiškienė 2021; Collivignarelli et al. 2020). In the UK, the concentrations of PM2.5 and PM10 were lower during the first wave of the pandemic, but during the second wave, they rose by 11.0%, compared to the prelockdown level in 2019 (Skirienė and Stasiškienė 2021; Jephcote et al. 2020).

It is a well-known fact that the level of air pollution can be influenced by seasonal sources, which dominate only in particular seasons and thus significantly contribute to air pollution. Tables S3 and S4 show that the concentrations of each pollutant correlate very well between years, reflecting seasonal changes with a similar trend observed in each of the studied years (Figs. 2b, 3b, 4b, 5b, 6b and 7b). Moreover, TCL does not show significant seasonal variations during the year (Table 2). Therefore, a strong increase in pollutant concentrations observed in the winter season may be attributed to the emission of pollutants from additional local sources, mainly stock emissions. In each studied year, the highest average monthly concentrations of pollutants were observed during the cold season (from November to March), and their values decreased reaching the minimum in June–August during the summer (warm) season (Figs. 2b, 3b, 4b, 5b, 6b and 7b). Based on these data, we calculated the average concentration of pollutants for the cold season and warm seasons in 2019–2021. We thus estimated vehicle traffic emission as average values in the warm season, and consequently, the differences between the values in cold and warm seasons as emissions related to stack emission. In TS-C, it is estimated that vehicle traffic, i.e., about 73,000 vehicles per day, may generate 30 µg/m^3^ of PM10, about 15 µg/m^3^ of PM2.5, and about 120 µg/m^3^ of NOx. By contrast, during the winter season, stack emissions can release about 67 µg/m^3^ of NOx, about 22 µg/m^3^ of PM2.5, and 30 µg/m^3^ of PM10 into the atmosphere. The comparison shows that during the winter season stack emissions contribute more to the air pollution level compared to combustion and non-combustion vehicle emissions. Besides the effect of combustion on local and distant emission, unfavorable geographical conditions for pollution dispersion, such as low natural ventilation in cities, wind speed, air temperature inversion, or katabatic flow in Cracow, can also contribute to a high level of PM2.5 in the cold half of the year. Hajto and Rowada (2010) indicated that the monthly frequency of stable atmospheric conditions in Cracow can vary from 81 to 74%, in March and October, respectively. During stable atmospheric conditions, there was an increase in PM concentration and the values were the highest.

For Warsaw, no significant changes in the concentrations of pollutants (Figs. 2b, 3b and 4b) in cold and warm seasons were observed as was evident for Cracow (Fig. 5b, 6b and 7b). In fact, only for PM2.5, a characteristic variation related to seasonal changes was observed, with high values in cold and low values in warm parts of the year. It can be estimated that about 20 µg/m^3^ of PM2.5 comes from sources other than vehicle traffic, which generates 10–20 µg/m^3^ of PM2.5 particles. This could be long-range emissions from the Warsaw beltway, the towns surrounding the city where, unlike the central districts of Warsaw, households are heated by coal-burning stoves. Another explanation may be the emission of fine PM from three power plants supplying heat and electricity to Warsaw. Although electrostatic precipitators are used to reduce particulate emissions, the finest particles still get into the atmosphere and particularly large quantities of these particles are observed when there is a high demand for heat, i.e., during the cold season.

## Conclusion

The study of the levels of NOx, PM2.5, and PM10 pollutants during forced lockdown allowed a better understanding of the impact of vehicle motion on changes in air quality at a local scale. The results showed a significant decrease in the concentration of NOx and PM2.5 in the atmosphere due to a reduction of emissions resulting from traffic.

Compared to 2019, we detected a significant reduction in the concentrations of pollutants during the period of full lockdown (April 2020): NOx by about 26%, PM2.5 by about 38%, and PM10 by about 21% in Warsaw; and NOx by about 45%, PM2.5 by about 13%, and PM10 by about 21% in Cracow. The main reason for the reduction of PM2.5, PM10, and NO_2_ was a significant decrease in local transportation, reducing the consumption of crude oil and gasoline, which greatly influences air quality.

In the years 2020–2021, during the COVID-19 pandemic, the average annual concentrations of NOx, PM2.5, and PM10, respectively, decreased by about 19%, about 19%, and about 18% in Warsaw and by about16%, about 22%, and about 2% in Cracow, compared to 2019 before the pandemic.

Seasonal changes (cold months versus warm months of the year) in average monthly concentrations of pollutants allowed estimating the contribution of traffic-related sources to the overall level of air pollution in the studied locations. It was estimated that about 30 µg/m^3^ of PM10, about 15 µg/m^3^ of PM2.5, and about 120 µg/m^3^ of NOx come from moving vehicles in Cracow with a traffic intensity of about 73,000 vehicles per day and 20 µg/m^3^ of PM2.5 come from vehicles in Warsaw with a traffic intensity of about 70,000 vehicles per day.

Based on the results of our study, we conclude that traffic-related exhaust emissions may be best reflected by NOx, as at both studied locations the NOx concentrations correlated well with TCLs. It is very likely that other local sources, mainly low emissions, contributing to PM2.5 generated by non-exhaust emissions disturbed or masked the relationship between this pollutant and vehicle intensity. This was clearly seen in Cracow, where local sources, mainly stack emissions conditioned by seasons of the year and the type of fuel used for apartment heating, were responsible for the weak correlations observed between TCL and PM2.5 concentration.

## Supplementary Information

Below is the link to the electronic supplementary material.

Supplementary file1 (DOCX 104 KB)

## Data Availability

The datasets generated during and/or analyzed during the current study are available from the corresponding author upon reasonable request.
