# Publisher Correction: Effect of COVID-19 pandemic restrictions on air pollution at a local scale in urban areas affected by high-intensity vehicle traffic in Poland

**DOI:** 10.1007/s11600-023-01026-3

**Published:** 2023-01-24

**Authors:** Beata Górka-Kostrubiec, Katarzyna Dudzisz

**Affiliations:** Institute of Geophysics, Polish Academy of Sciences, Księcia Janusza 64, 01-452 Warsaw, Poland

**Correction to: Acta Geophysica** https://doi.org/10.1007/s11600-022-01005-0

The publication of this article unfortunately contained mistakes. The corrections in figure legends 3 and 4 were not carried out from typesetter. The corrected legends are given below.

Fig. 3 **a** Average diurnal concentrations of PM2.5 prior to and after a lockdown in 2020 (light area) and comparison for the same period in 2019 (dark area). **b** Average monthly concentrations of PM2.5 in 2021, 2020, and 2019. Data from air pollution traffic monitoring station (TS-W), Warsaw, Poland. B-spline curve (basis spline function) was applied for the smoothness of experimental data

Fig. 4 **a** Average diurnal concentrations of PM10 prior to and after a lockdown in 2020 (light area) and comparison for the same period in 2019 (dark area). **b** Average monthly concentrations of PM10 in 2021, 2020, and 2019. Data from air pollution traffic monitoring station (TS-W), Warsaw, Poland. B-spline curve (basis spline function) was applied for the smoothness of experimental data

The original article has been corrected.

