# Supplementary material for: Effect of COVID-19 pandemic restrictions on air pollution at a local scale in urban areas affected by high-intensity vehicle traffic in Poland
Source: Acta Geophys. 2022 Dec 30;71(2):1085–97. doi: 10.1007/s11600-022-01005-0 (PMC9802015; doi:10.1007/s11600-022-01005-0)
Supplement: Supplementary file 1 — Supplementary file1 (DOCX 104 KB) [file 11600_2022_1005_MOESM1_ESM.docx]

Supplementary Material

Effect of COVID-19 pandemic restrictions on air pollution at a local scale in urban areas affected by high-intensity vehicle traffic in Poland.

Beata Górka-Kostrubiec and Katarzyna Dudzisz

**Table S1.** Stations belonging to the national air quality monitoring network in Warsaw and Cracow, Poland (Chief Inspectorate for Environmental Protection, <http://powietrze.gios.gov.pl/pjp/archives>), with their attributes, i.e. country code, location, type of station, type of area, type of measurement type and type of monitored pollutants.

| ***Country code*** | ***Location*** | ***Type of station*** | ***Type of area*** | ***Type of measurement*** | ***Pollutants*** |
| --- | --- | --- | --- | --- | --- |
| **Warsaw** | | | | | |
| **MzWarAlNiepo *** | Niepodległości  avenue | traffic | urban | automatic | NO, NOx, NO2, PM10, PM2.5 |
| **MzWarChrosci** | Chruścickiego  street | background | urban | automatic | NO, NOx, NO2, PM10, PM2.5 |
| **MzWarWokalna** | Wokalna street | background | urban | automatic | NO, NOx, NO2, PM10, PM2.5 |
| **MzWarKondrat** | Kondratowicza  street | background | urban | automatic | NO, NOx, NO2, PM10, PM2.5 |
| **MzWarBajkowa** | Bajkowa street | background | urban | automatic | PM10, PM2.5 |
| **MzWarTolstoj** | Tołstoja street | background | urban | automatic | PM10, PM2.5 |
| **MzWarAKrzywo** | Anieli Krzywoń  street | background | urban | manual | PM10 |
| **Cracow** | | | | | |
| **MpKrakAlKras **** | Krasińskiego  avenue | traffic | urban | automatic | NO, NOx, NO2, PM10, PM2.5 |
| **MpKrakDietla** | Dietla street | traffic | urban | automatic | NO, NOx, NO2, PM10 |
| **MpKrakBulwar** | Bulwarowa street | industrial | urban | automatic | NO, NOx, NO2, PM10, PM2.5 |
| **MpKrakWadow** | Wadów estate | industrial | urban | automatic | PM10 |
| **MpKrakZloRog** | Złoty Róg street | background | urban | automatic | PM10 |
| **MpKrakBujaka** | Bujaka street | background | urban | automatic | NO, NOx, NO2, PM10, PM2.5 |
| **MpKrakOsPias** | Oś Piastów | background | urban | automatic | PM10, PM2.5 |

* used in this study (TS-W), ** used in this study (TS-C)

**Table S2.** Average temperature, humidity, precipitation, wind speed, wind dominant direction and haze in March and April 2019, 2020 and 2021, in Warsaw and Cracow.

|  | ***Warsaw*** | | | | | | ***Cracow*** | | | | | |
| --- | --- | --- | --- | --- | --- | --- | --- | --- | --- | --- | --- | --- |
| year | 2019 | | 2020 | | 2021 | | 2019 | | 2020 | | 2021 | |
| month | III | IV | III | IV | III | IV | III | IV | III | IV | III | IV |
| temperature (°C) | 6.1 | 10.6 | 5.0 | 9.5 | 3.3 | 7.0 | 6.2 | 10.0 | 4.9 | 9.5 | 3.4 | 6.2 |
| humidity (%) | 68 | 53 | 59 | 48 | 72 | 69 | 66 | 65 | 65 | 52 | 68 | 72 |
| precipitation (mm) | 27.9 | 3.2 | 13.0 | 7.5 | 18.3 | 58.2 | 23.9 | 72.7 | 15.2 | 4.4 | 18.2 | 65.5 |
| wind speed (m/s) | 4.1 | 3.4 | 3.3 | 3.2 | 3.3 | 3.5 | 2.1 | 2.1 | 2.1 | 1.6 | 1.7 | 2.0 |
| wind direction (dominant) | W, S | E | ^(1)^ | ^(1)^ | ^(1)^ | ^(1)^ | W | NE, NW | W, E | W | W | W |
| haze (hours) | N/D | 0 | 0 | 0 | 7.5 | 8.5 | N/D | 0 | N/D | N/D | N/D | 0 |

^(1)^ changing direction of wind; N/D – no data given.

**Table S3.** The correlations between pollutant concentrations and intensity of vehicle traffic, expressed as average traffic congestion level (TCL) 2019, 2020 and 2021, at the studied locations in Warsaw (Traffic Station Warsaw; TS-W)

|  |  | ***PM2.5*** | | | ***PM10*** | | | ***NOx*** | | |
| --- | --- | --- | --- | --- | --- | --- | --- | --- | --- | --- |
|  |  | 2019 | 2020 | 2021 | 2019 | 2020 | 2021 | 2019 | 2020 | 2021 |
| ***PM2.5*** | 2019 |  | 0.74 |  | 0.79 |  |  | 0.43 |  |  |
|  | 2020 |  |  | 0.54 |  | 0.47 |  |  | 0.2 |  |
|  | 2021 | 0.88 |  |  |  |  | 0.67 |  |  | 0.19 |
| ***PM10*** | 2019 | 0.79 |  |  |  | 0.41 |  | 0.48 |  |  |
|  | 2020 |  | 0.47 |  |  |  | 0.27 |  | -0.29 |  |
|  | 2021 |  |  | 0.67 | 0.72 |  |  |  |  | 0.10 |
| ***NOx*** | 2019 | 0.43 |  |  | 0.48 |  |  |  | 0.64 |  |
|  | 2020 |  | 0.2 |  |  | -0.29 |  |  |  | 0.54 |
|  | 2021 |  |  | 0.19 |  |  | 0.10 | 0.36 |  |  |

**Table S4.** The correlations between pollutant concentrations and intensity of vehicle traffic, expressed as average traffic congestion level (TCL) 2019, 2020 and 2021, at the studied locations in Cracow (Traffic Station Traffic Station Cracow; TS-C)

|  |  | ***PM2.5*** | | | | | ***PM10*** | | | | | ***NOx*** | | | | | |  |
| --- | --- | --- | --- | --- | --- | --- | --- | --- | --- | --- | --- | --- | --- | --- | --- | --- | --- | --- |
|  |  | 2019 | | 2020 | 2021 | | 2019 | | 2020 | 2021 | | 2019 | | 2020 | | 2021 | |  |
| ***PM2.5*** | 2019 | |  | 0.73 |  | 0.97 | |  | | |  | | 0.67 | |  | |  | |
|  | 2020 | |  |  | 0.52 |  | | 0.99 | | |  | |  | | 0.73 | |  | |
|  | 2021 | | 0.89 |  |  |  | |  | | | 0.97 | |  | |  | | 0.74 | |
| ***PM10*** | 2019 | | 0.97 |  |  |  | | 0.71 | | |  | | 0.74 | |  | |  | |
|  | 2020 | |  | 0.99 |  |  | |  | | | 0.64 | |  | | 0.74 | |  | |
|  | 2021 | |  |  | 0.97 | 0.90 | |  | | |  | |  | |  | | 0.75 | |
| ***NOx*** | 2019 | | 0.67 |  |  | 0.74 | |  | | |  | |  | | 0.71 | |  | |
|  | 2020 | |  | 0.72 |  |  | | 0.74 | | |  | |  | |  | | 0.82 | |
|  | 2021 | |  |  | 0.70 |  | |  | | | 0.75 | | 0.67 | |  | |  | |


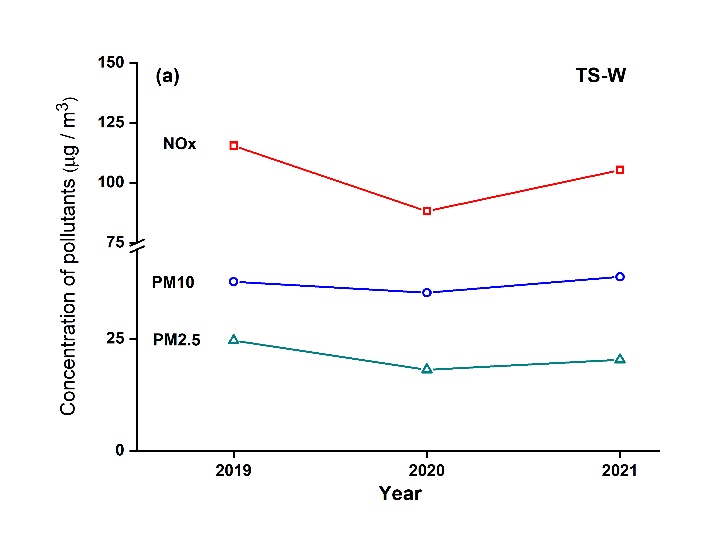

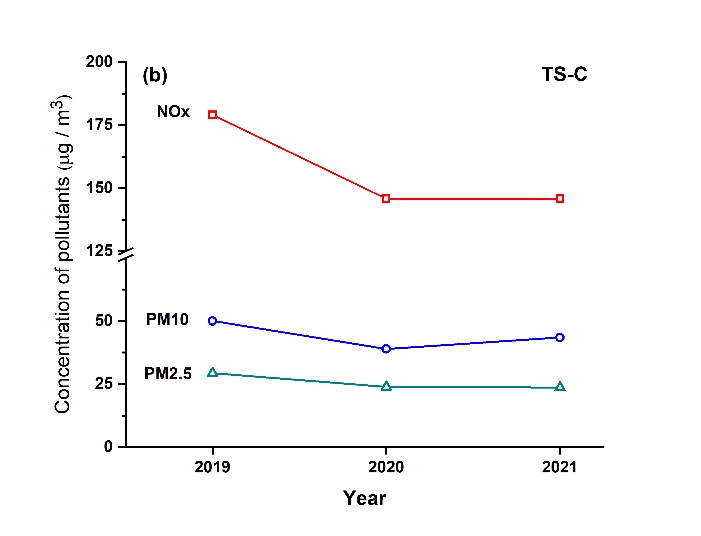
**Fig. S1.** Annual average concentrations of pollutant in 2019, 2020, and 2021 in the study area TS-W, Warsaw (a) and TS-C, Cracow (b).
